# Firing rate equations require a spike synchrony mechanism to correctly describe fast oscillations in inhibitory networks

**DOI:** 10.1371/journal.pcbi.1005881

**Published:** 2017-12-29

**Authors:** Federico Devalle, Alex Roxin, Ernest Montbrió

**Affiliations:** 1 Center for Brain and Cognition, Department of Information and Communication Technologies, Universitat Pompeu Fabra, Barcelona, Spain; 2 Department of Physics, Lancaster University, Lancaster, United Kingdom; 3 Centre de Recerca Matemàtica, Campus de Bellaterra, Edifici C, Bellaterra, Barcelona, Spain; Institute for Neuroscience and Medicine (INM-6), GERMANY

## Abstract

Recurrently coupled networks of inhibitory neurons robustly generate oscillations in the gamma band. Nonetheless, the corresponding Wilson-Cowan type firing rate equation for such an inhibitory population does not generate such oscillations without an explicit time delay. We show that this discrepancy is due to a voltage-dependent spike-synchronization mechanism inherent in networks of spiking neurons which is not captured by standard firing rate equations. Here we investigate an exact low-dimensional description for a network of heterogeneous canonical Class 1 inhibitory neurons which includes the sub-threshold dynamics crucial for generating synchronous states. In the limit of slow synaptic kinetics the spike-synchrony mechanism is suppressed and the standard Wilson-Cowan equations are formally recovered as long as external inputs are also slow. However, even in this limit synchronous spiking can be elicited by inputs which fluctuate on a time-scale of the membrane time-constant of the neurons. Our meanfield equations therefore represent an extension of the standard Wilson-Cowan equations in which spike synchrony is also correctly described.

## Author summary

Population models describing the average activity of large neuronal ensembles are a powerful mathematical tool to investigate the principles underlying cooperative function of large neuronal systems. However, these models do not properly describe the phenomenon of spike synchrony in networks of neurons. In particular, they fail to capture the onset of synchronous oscillations in networks of inhibitory neurons. We show that this limitation is due to a voltage-dependent synchronization mechanism which is naturally present in spiking neuron models but not captured by traditional firing rate equations. Here we investigate a novel set of macroscopic equations which incorporate both firing rate and membrane potential dynamics, and that correctly generate fast inhibition-based synchronous oscillations. In the limit of slow-synaptic processing oscillations are suppressed, and the model reduces to an equation formally equivalent to the Wilson-Cowan model.

## Introduction

Since the seminal work of Wilson and Cowan [1], population models of neuronal activity have become a standard tool of analysis in computational neuroscience. Rather than focus on the microscopic dynamics of neurons, these models describe the collective properties of large numbers of neurons, typically in terms of the mean firing rate of a neuronal ensemble. In general, such population models, often called firing rate equations, cannot be exactly derived from the equations of a network of spiking neurons, but are obtained using heuristic mean-field arguments, see e.g. [2–6]. Despite their heuristic nature, heuristic firing rate equations (which we call H-FRE) often show remarkable qualitative agreement with the dynamics in equivalent networks of spiking neurons [7–10], and constitute an extremely useful modeling tool, see e.g. [11–28]. Nonetheless, this agreement can break down once a significant fraction of the neurons in the population fires spikes synchronously, see e.g. [29]. Such synchronous firing may come about due to external drive, but also occurs to some degree during spontaneously generated network states.

As a case in point, here we focus on partially synchronized states in networks of heterogeneous inhibitory neurons. Inhibitory networks are able to generate robust macroscopic oscillations due to the interplay of external excitatory inputs with the inhibitory mean field produced by the population itself. Fast synaptic processing coupled with subthreshold integration of inputs introduces an effective delay in the negative feedback facilitating the emergence of what is often called Inter-Neuronal Gamma (ING) oscillations [30–38]. Modeling studies with networks of spiking neurons demonstrate that, in heterogeneous inhibitory networks, large fractions of neurons become frequency-entrained during these oscillatory episodes, and that the oscillations persist for weak levels of heterogeneity [30, 32, 34]. Traditional H-FRE (also referred to as Wilson-Cowan equations) fail to describe such fast oscillations. To overcome this limitation, explicit fixed time delays have been considered in H-FRE as a heuristic proxy for the combined effects of synaptic and subthreshold integration [9, 10, 36, 39].

Here we show that fast oscillations in inhibitory networks are correctly described by a recently derived set of exact macroscopic equations for quadratic integrate-and-fire neurons (that we call QIF-FRE) which explicitly take into account subthreshold integration [40]. Specifically, the QIF-FRE reveal how oscillations arise via a voltage-dependent spike synchronization mechanism, missing in H-FRE, as long as the recurrent synaptic kinetics are sufficiently fast. In the limit of slow recurrent synaptic kinetics intrinsically generated oscillations are suppressed, and the QIF-FRE reduce to an equation formally identical to the Wilson-Cowan equation for an inhibitory population. However, even in this limit, fast fluctuations in external inputs can drive transient spike synchrony in the network, and the slow synaptic approximation of the QIF-FRE breaks down. This suggests that, in general, a correct macroscopic description of spiking networks requires keeping track of the mean subthreshold voltage along with the mean firing rate.

Additionally, the QIF-FRE describe the disappearance of oscillations for sufficiently strong heterogeneity which is robustly observed in simulations of spiking networks. Finally, we also show that the phase diagrams of oscillatory states found in the QIF-FRE qualitatively match those observed in simulations of populations of more biophysically inspired Wang-Buzsáki neurons [30]. This shows that not only are the QIF-FRE an exact mean-field description of networks of heterogeneous QIF neurons, but that they also provide a qualitatively accurate description of dynamical states in networks of spiking neurons more generally, including states with significant spike synchrony.

## Results

**Fig 1:**
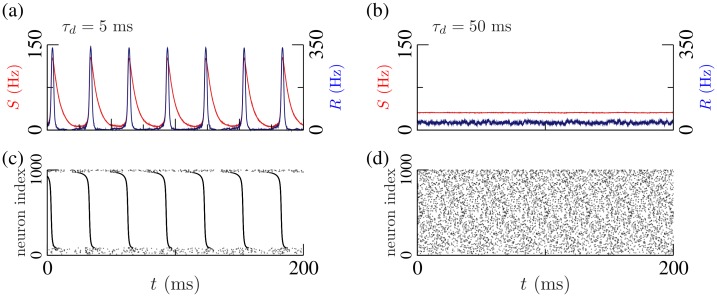
Networks of heterogeneous inhibitory neurons with fast synaptic kinetics (*τ*_*d*_ = 5 ms) display macroscopic oscillations in the gamma range (ING oscillations) due to collective synchronization. Panels (a) and (c) show the time series of the synaptic variable *S* (red) and mean firing rate *R* (blue), and the raster plot of a population of *N* = 1000 inhibitory Wang-Buzsáki neurons [30] with first order fast synaptic kinetics. The oscillations are suppressed considering slow inhibitory synapses (*τ*_*d*_ = 50 ms), as shown in Panels (b) and (d). See Materials and methods for details on the numerical simulations.

Recurrent networks of spiking neurons with inhibitory interactions readily generate fast oscillations. Fig 1 shows an illustration of such oscillations in a network of globally coupled Wang-Buzsáki (WB) neurons [30]. Panels (a,c) show the results of a numerical simulation of the network for fast synapses (time constant *τ*_*d*_ = 5 ms), compared to the membrane time constant of the neuron model (*τ*_*m*_ = 10 ms). Although the neurons have different intrinsic frequencies due to a distribution in external input currents, the raster plot reveals that fast inhibitory coupling produces the frequency entrainment of a large fraction of the neurons in the ensemble. This collective synchronization is reflected at the macroscopic scale as an oscillation with the frequency of the synchronous cluster of neurons [41, 42]. Indeed, panel (a) shows the time series of both the mean synaptic activation variable *S*, and the mean firing rate *R*, which display ING oscillations. Panels (b,d) of Fig 1 show the disappearance of the synchronous state when the synaptic kinetics is slow (*τ*_*d*_ = 50 ms).

### A heuristic firing rate equation

A heuristic firing rate description of the spiking network simulated in Fig 1 takes the form [1, 5]

$$\begin{array}{ccc} \tau_{m}\dot{R} & = & -R+\Phi (-J\tau_{m}S+\Theta ), \end{array}$$ (1a)

$$\begin{array}{ccc} \tau_{d}\dot{S} & = & -S+R. \end{array}$$ (1b)

where *R* represents the mean firing rate in the population, *S* is the synaptic activation, and the time constants *τ*_*m*_ and *τ*_*d*_ are the neuronal and synaptic time constants respectively [39, 43]. The input-output function *Φ*, also known as the f-I curve, is a nonlinear function, the form of which depends on the details of the neuronal model and on network parameters. Finally, *J* ≥ 0 is the synaptic strength and Θ is the mean external input current compared to threshold. In contrast with the network model, the H-FRE Eq (1) cannot generate sustained oscillations. In fact, a linear stability analysis of steady state solutions in Eq (1) shows that the resulting eigenvalues are

$$\begin{array}{c} \lambda =-\alpha (1\pm \sqrt{1-\beta }), \end{array}$$ (2)

where the parameter *α* = (*τ*_*m*_ + *τ*_*d*_)/(2*τ*_*m*_ *τ*_*d*_) is always positive. Additionally, the parameter *β* = [4*τ*_*m*_ *τ*_*d*_(1 + *Jτ*_*m*_ Φ′)]/(*τ*_*m*_ + *τ*_*d*_)^2^ is also positive, since the f-I curve Φ(*x*) is an increasing function, and its derivative evaluated at the steady state is then Φ′ > 0. Therefore the real part of the eigenvalue λ is always negative and hence steady states are always stable, although damped oscillations are possible, e.g. for strong enough coupling *J*. Introducing an explicit fixed time delay in Eq (1) can lead to the generation of oscillations with a period on the order of about twice the delay [9, 10, 36]. Nonetheless, inhibitory networks of spiking neurons robustly show oscillations even in the absence of explicit delays, as seen in Fig 1. This suggests that there is an additional mechanism in the network dynamics, key for driving oscillatory behavior, which H-FRE do not capture.

### An exact firing rate equation which captures spike synchrony

Here we show that the mechanism responsible for the generation of the oscillations shown in Fig 1 is the interplay between the mean firing rate and membrane potential, the dynamics of which reflect the degree of spike synchrony in the network. To do this, we use a set of exact macroscopic equations which have been recently derived from a population of heterogeneous quadratic integrate-and-fire (QIF) neurons [40]. We refer to these equations as the QIF-FRE. The QIF-FRE with exponential synapses have the form

$$\begin{array}{ccc} \tau_{m}\dot{R} & = & \frac{\Delta }{\pi \tau_{m}}+2RV, \end{array}$$ (3a)

$$\begin{array}{ccc} \tau_{m}\dot{V} & = & V^{2}-{\left(\pi \tau_{m}R\right)}^{2}-J\tau_{m}S+\Theta , \end{array}$$ (3b)

$$\begin{array}{ccc} \tau_{d}\dot{S} & = & -S+R. \end{array}$$ (3c)

where Δ is a parameter measuring the degree of heterogeneity in the network and the other parameters are as in the H-FRE Eq (1). Eq (3) are an exact macroscopic description of the dynamics in a large network of heterogeneous QIF neurons with inhibitory coupling. In contrast with the traditional firing rate equations Eq (1), they contain an explicit dependence on the subthreshold state of the network, and hence capture not only macroscopic variations in firing rate, but also in spike synchrony. Specifically, a transient depolarizing input which drives the voltage to positive values (the voltage has been normalized such that positive values are suprathrehsold, see Materials and methods) will lead to a sharp growth in the firing rate through the bilinear term in Eq (3a). Simulations in the corresponding network model reveal that this increase is due to the synchronous spiking of a subset of neurons [40]. This increase in firing rate leads to a hyperpolarization of the mean voltage through the quadratic term in *R* in Eq (3b). This term describes the effect of the neuronal reset. This decrease in voltage in turn drives down the mean firing rate, and the process can repeat. Therefore the interplay between mean firing rate and mean voltage in Eq (3) can generate oscillatory behavior; this behavior corresponds to transient bouts of spike synchrony in the spiking network model.

It is precisely the latency inherent in this interplay which provides the effective delay, which when coupled with synaptic kinetics, generates self-sustained fast oscillations. In fact, in the limit of instantaneous synapses (*τ*_*d*_ → 0), Eq (3) robustly display damped oscillations due to the spike generation and reset mechanism described in the preceding paragraph [40]. Contrast this with the dynamics in Eq (1) in the same limit: the resulting H-FRE is one dimensional and hence cannot oscillate.

On the face of things the Eq (3) appear to have an utterly distinct functional form from the traditional Wilson-Cowan Eq (1). In particular, the f-I curve in H-FRE traditionally exhibits an expansive nonlinearity at low rates and a linearization or saturation at high rates, e.g. a sigmoid. There is no such function visible in the QIF-FRE which have only quadratic nonlinearities. However, this seeming inconsistency is simply due to the explicit dependence of the steady-state f-I curve on the subthreshold voltage in Eq (3). In fact, the steady-state f-I curve in the QIF-FRE is “typical” in the qualitative sense described above. Specifically, solving for the steady state value of the firing rate in Eq (3) yields

$$\begin{array}{c} R_{*}=\Phi \left(-J\tau_{m}R_{*}+\Theta \right), \end{array}$$ (4)

where

$$\begin{array}{c} \Phi \left(I\right)=\frac{1}{\sqrt{2}\pi \tau_{m}}\sqrt{I+\sqrt{I^{2}+\Delta^{2}}}. \end{array}$$ (5)

**Fig 2:**
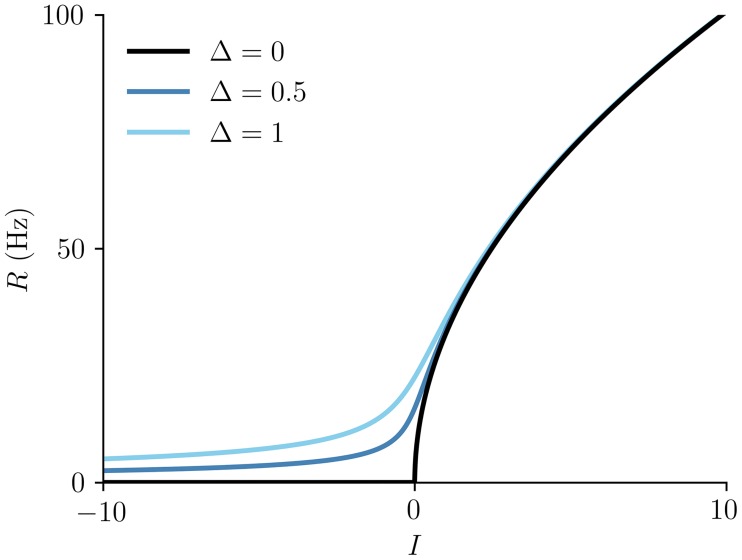
The f-I curve Φ(*I*), Eq (5), for several values of the heterogeneity parameter Δ. The membrane time constant is *τ*_*m*_ = 10ms.

The f-I curve Eq (5) is shown in Fig 2 for several values of the parameter Δ, which measures the degree of heterogeneity in the network. Therefore, the steady-state firing rate in Eq (3), which corresponds exactly to that in a network of heterogeneous QIF neurons, could easily be captured in a heuristic model such as Eq (1) by taking the function Φ to have the form as in Eq (5). On the other hand, the non-steady behavior in Eq (3), and hence in spiking networks as well, can be quite different from that in the heuristic Eq (1).

#### Fast oscillations in the QIF-FRE

**Fig 3:**
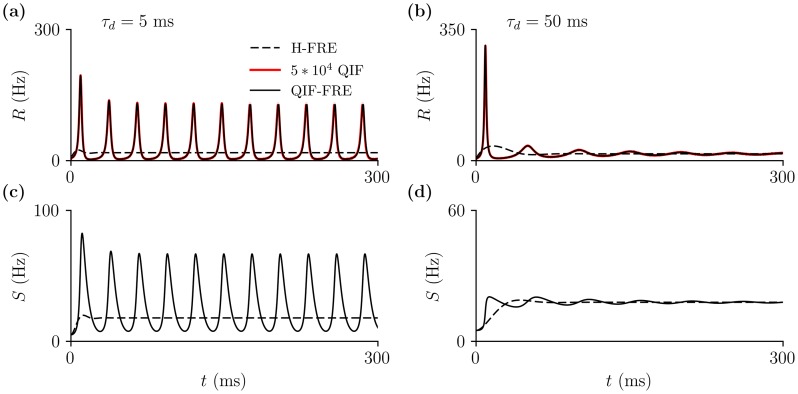
Heuristic FRE Eq (1) do not display inhibition-based fast oscillations. In contrast, networks of QIF neurons (red) and their corresponding QIF-FRE Eq (3) (solid black) do show ING oscillations for fast synaptic kinetics (*τ*_*d*_ = 5 ms). These oscillations are suppressed for slow synaptic kinetics (*τ*_*d*_ = 50 ms), as in the Wang-Buzsáki model shown in Fig 1. Panels (a,b) show the times series of the Firing Rate variable *R* of the FRE models, as well as the mean firing rate of a population of *N* = 5 × 10^4^ QIF neurons (red). Panels (c,d) show the time series of the synaptic *S* variables for the H-FRE (dashed line) and QIF-FRE (solid line). Parameters: *τ*_*m*_ = 10 ms, *J* = 21, Θ = 4, Δ = 0.3. Initial values: *R*(0) = *S*(0) = 5 Hz, *V*(0) = 0.

We have seen that decreasing the time constant of synaptic decay *τ*_*d*_ in a network of inhibitory spiking neurons lead to sustained fast oscillations, while such a transition to oscillations is not found in the heuristic rate equations, in which the synaptic dynamics are taken into account Eq (1). The exact QIF-FRE, on the other hand, do generate oscillations in this regime. Fig 3 shows a comparison of the firing rate *R* and synaptic variable *S* from simulations of the QIF-FRE Eq (3), with that of the H-FRE Eq (1), for two different values of the synaptic time constants. Additionally, we also performed simulations of a network of *N* = 5 × 10^4^ QIF neurons. The mean firing rate of the network is shown in red, and perfectly agrees with the firing rate of the low dimensional QIF-FRE (solid black lines). The function Φ in Eq (1) is chosen to be that from Eq (5), which is why the firing rate from both models converges to the same steady state value when oscillations are not present (panels (b,d) for *τ*_*d*_ = 50 ms). We will study the transition to fast oscillations in Eq (3) in greater details in the following sections.

### Linear stability analysis of the QIF-FRE

We can investigate the emergence of sustained oscillations in Eq (3) by considering small amplitude perturbations of the steady-state solution. If we take *R* = R_*_ + *δRe*^*λt*^, *V* = V_*_ + *δVe*^*λt*^ and *S* = S_*_ + *δSe*^*λt*^, where *δR*, *δV*, *δS* ≪ 1, then the sign of the real part of the eigenvalue λ determines whether the perturbation grows or not. Plugging this ansatz into Eq (3) yields three coupled linear equations which are solvable if the following characteristic equation also has a solution

$$\begin{array}{c} -2J\tau_{m}R_{*}=\left(1+\tau_{d}\lambda \right)\left[{\left(2\pi \tau_{m}R_{*}\right)}^{2}+{\left(\tau_{m}\lambda +\frac{\Delta }{\pi \tau_{m}R_{*}}\right)}^{2}\right]. \end{array}$$ (6)

**Fig 4:**
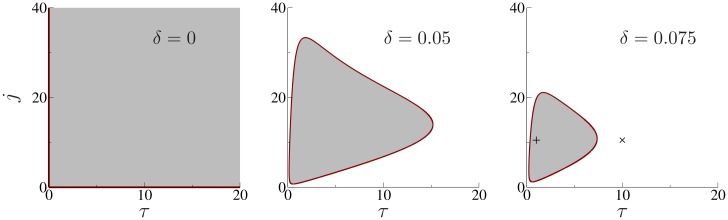
The ratio of the width to the center of the distribution of currents Eq (14), *δ* = Δ/Θ, determines the presence of fast oscillations in the QIF-FRE. Oscillations disappear above the critical value given by Eq (8). The panels show the Hopf boundaries of QIF-FRE with first-order synapses, for different values of *δ*, obtained solving the characteristic Eq (6) with Re(λ) = 0, see Materials and methods. Shaded regions are regions of oscillations. Symbols in the right panel correspond to the parameters used in Fig 3.

The left hand side of Eq (6) is always negative and, for *τ*_*d*_ = 0, this implies that the solutions λ are necessarily complex. Hence, for instantaneous synapses, the fixed point of the QIF-FRE is always of focus type, reflecting transient episodes of spike synchrony in the neuronal ensemble [40]. In contrast, setting *τ*_*d*_ = 0 in the H-FRE, the system becomes first order and oscillations are not possible. This is the critical difference between the two firing rate models. In fact, and in contrast with the eigenvalues Eq (2) corresponding to the growth rate of small perturbations in the H-FRE, here oscillatory instabilities may occur for nonvanishing values of *τ*_*d*_. Fig 4 shows the Hopf boundaries obtained from Eq (6), as a function of the normalized synaptic strength $j=J/\sqrt{\Theta }$ and the ratio of the synaptic and neuronal time constants $\tau =\sqrt{\Theta }\tau_{d}/\tau_{m}$, and for different values of the ratio *δ* = Δ/Θ —see Materials and methods, Eqs (19)–(21). The shaded regions correspond to parameter values where the QIF-FRE display oscillatory solutions.

#### Identical neurons

In the simplest case of identical neurons we find the boundaries of oscillatory instabilities explicitly. Indeed, substituting λ = *ν* + *iω* in Eq (6) we find that, near criticality (|*ν*| ≪ 1), the real part of the eigenvalue is

$$\begin{array}{c} \nu \approx J\tau \frac{R_{*}}{1+{\left(2\pi \tau_{d}R_{*}\right)}^{2}}. \end{array}$$ (7)

Thus, the fixed point (4) is unstable for *Jτ* > 0, and changes its stability for either *J* = 0, or *τ* = 0. In particular, given a non-zero synaptic time constant there is an oscillatory instability as the sign of the synaptic coupling *J* changes from positive to negative. Therefore oscillations occur only for inhibitory coupling [44–46]. The frequency along this Hopf bifurcation line is determined entirely by the intrinsic firing rate of individual cells *ω*_*c*_ = 2πR_*_.

On the other hand, in the limit of fast synaptic kinetics, i.e. *τ*_*d*_ = 0 in Eq (6), we find another Hopf bifurcation with $\omega_{c}=\frac{1}{\tau_{m}}\sqrt{2\tau_{m}R_{*}\left(J+2\pi^{2}\tau_{m}R_{*}\right)}$. This reflects the fact that oscillations cannot be induced if the synaptic interactions are instantaneous. The left panel of Fig 4 shows the phase diagram with the Hopf boundaries depicted in Red, reflecting that oscillations are found for all values of inhibitory coupling and for non-instantaneous synaptic kinetics.

#### Heterogeneous neurons

Once heterogeneity is added to the network the region of sustained oscillatory behavior shrinks, see Fig 4, center and right. The red closed curves correspond to the Hopf bifurcations, which have been obtained in parametric form from the characteristic eq (6), see Materials and methods. Note that for small levels of *δ*, oscillations are present in a closed region of the phase diagram, and disappear for large enough values of *τ* (the synaptic time constant relative to the neuronal time constant). Further increases in *δ* gradually reduce the region of oscillations until it fully disappears at the critical value

$$\begin{array}{c} \delta_{c}={\left(\frac{\Delta }{\Theta }\right)}_{c}=\frac{1}{5}\sqrt{5-2\sqrt{5}}=0.1453\ldots , \end{array}$$ (8)

which has been obtained analytically from the characteristic Eq (6), see Materials and methods. This result is consistent with numerical studies investigating oscillations in heterogeneous inhibitory networks which indicate that gamma oscillations are fragile against the presence of quenched heterogeneity [30, 32, 34].

**Fig 5:**
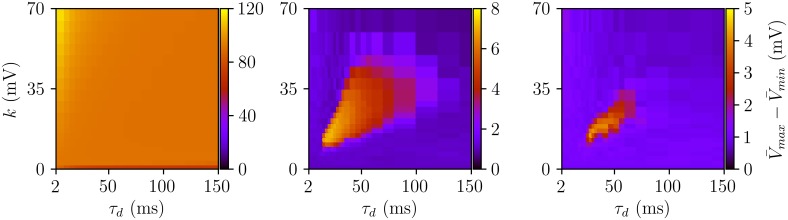
Amplitude of the oscillations of the mean membrane potential for a population of *N* = 1000 WB neurons. From left to right: $\delta =\sigma /\bar{I}=0$, 0.05 and 0.06. Central and Right panels have *σ* = 0.01 *μ*A/cm^2^. See Materials and methods for details.

In the following, we compare the phase diagrams of Fig 4 with numerical results using heterogeneous ensembles of Wang-Buzsáki neurons with first order synapses. Instead of using the population mean firing rate or mean synaptic activation, in Fig 5 we computed the amplitude of the population mean membrane potential. This variable is less affected by finite-size fluctuations and hence the regions of oscillations are more easily distinguishable. The results are summarized in Fig 5 for different values of *δ* and have been obtained by systematically increasing the coupling strength *k* for fixed values of *τ*_*d*_. The resulting phase diagrams qualitatively agree with those shown in Fig 4. As predicted by the QIF-FRE, oscillations are found in a closed region in the (*τ*_*d*_, *k*) parameter space, and disappear for large enough values of *δ*. Here, the critical value of $\delta =\sigma /\bar{I}$ is about 6%, smaller than the critical value given by Eq (8). This is due to the steep f-I curve of the WB model, which implies a larger dispersion in the firing rates of the neurons even for small heterogeneities in the input currents.

Additionally, for small *τ*_*d*_ (fast synaptic kinetics) and strong coupling *k*, we observed small regions where the oscillations coexist with the asynchronous state —not shown. Numerical simulations indicate that this bistability is not present in the QIF-FRE. For strong coupling, and coexisting with the asynchronous state, we also observed various clustering states, already reported in the original paper of Wang & Buzsáki [30]. Clustering in inhibitory networks has also been observed in populations of conductance-based neurons with spike adaptation [47] or time delays [48]. The fact that such states do not emerge in the model Eq (12) may be due to the purely sinusoidal shape of the phase resetting curve of the QIF model [49–54].

### Firing rate equations in the limit of slow synapses

We have seen that the oscillations which emerge in inhibitory networks for sufficiently fast synaptic kinetics are correctly described by the firing rate equations Eq (3), but not by the heuristic Eq (1). The reason for this is that the oscillations crucially depend on the interaction between the population firing rate and the subthreshold membrane potential during spike initiation and reset; this interaction manifests itself at the network level through spike synchrony. Therefore, if one could suppress the spike synchrony mechanism, and hence the dependence on the subthreshold membrane potential, in Eq (3), the resulting equations ought to bear resemblance to Eq (1). In fact, as we saw in Fig 3, the two firing rate models become more similar when the synaptic kinetics become slower.

Next we show that the two models become identical, formally, in the limit of slow synaptic kinetics. To show this, we assume the synaptic time constant is slow, namely $\tau_{d}={\bar{\tau }}_{d}/\epsilon$ where 0 < *ϵ* ≪ 1, and rescale time as $\bar{t}=\epsilon t$. In this limit we are tracking the slow synaptic dynamics in while the neuronal dynamics are stationary to leading order, i.e.

$$\begin{array}{c} R_{*}=\Phi \left(-J\tau_{m}S+\Theta \right). \end{array}$$ (9)

Therefore, the dynamics reduce to the first order equation

$$\begin{array}{c} \tau_{d}\dot{S}=-S+\Phi \left(-J\tau_{m}S+\Theta \right). \end{array}$$ (10)

Notably, this shows that the QIF-FRE Eq (3), and the H-FRE (1), do actually have the same dynamics in the limit of slow synapses. In other words, Eq (10) is formally equivalent to the Wilson-Cowan equations for a single inhibitory population, and this establishes a mathematical link between the QIF-FRE and Heuristic firing rate descriptions. Additionally, considering slow second order synaptic kinetics (not shown), allows one to reduce the QIF-FRE with either alpha or double exponential synapses to a second-order system that formally corresponds to the so-called neural mass models largely used for modeling EEG data, see e.g. [6, 55–58].

#### External inputs and breakdown of the slow-synaptic limit Eq (10)

It is important to note that, in the derivation of Eq (10) we considered external inputs Θ to be constant. Then, if synapses are slow, the neuronal variables (*R* in the case of Eq (1) and *R* and *V* in the case of Eq (3)) decay rapidly to their fixed point values. However even in the limit of slow synapses, such reduction can break down if external inputs are time-varying Θ = Θ(*t*), with a time-scale which itself is not sufficiently slow.

**Fig 6:**
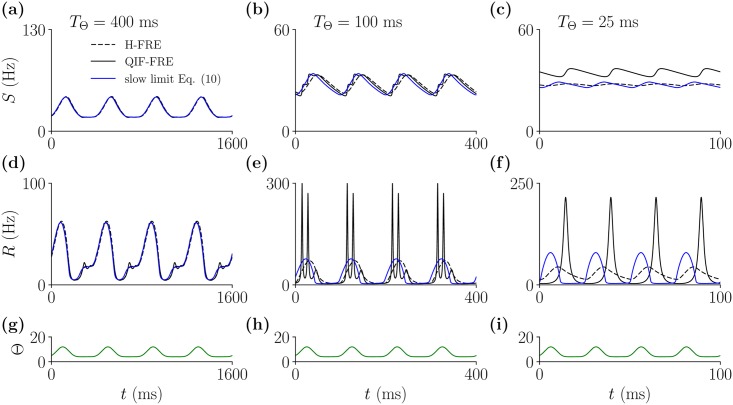
The reduction of the QIF-FRE to Eq (10) breaks down when neurons receive time-varying inputs. Panels (a-c): *S*-variable time series for QIF-FRE (solid Black), H-FRE (dashed Black) and Eq (10) (Blue), for decreasing values of the period *T*_Θ_ of the external periodic forcing Θ(*t*) = 4 + [1 + sin(2*πt*/*T*_*θ*_)]^3^ —shown in panels (g-i). In all cases, the synaptic time constant is slow *τ*_*d*_ = 100 ms, compared to the membrane time constant *τ*_*m*_ = 10 ms. Panels (d-f): *R*-variable time series. In the case of model Eq (10), the firing rate has been evaluated using Eq (9). Other parameters are *J* = 21, Δ = 0.3.

To demonstrate this, in Fig 6, we compared the dynamics of the QIF-FRE and H-FRE with the approximation Eq (10), for periodic stimuli of various periods —panels (g-i)—, and always considering slow synapses, *τ*_*d*_ = 100 ms. As expected, the models show good agreement for very slow external inputs —see panels (a,d)—, but this discrepancy is strongly magnified for fast external inputs Specifically, for fast inputs —see panels (c,f)—, the dynamics of the *S* and *R* variables of the QIF-FRE are clearly different form those of the other models. This illustrates that even in the limit of slow synapses, the response of spiking networks to arbitrary time-varying inputs will always generate some degree of spike synchrony.

## Discussion

Firing rate models, describing the average activity of large neuronal ensembles are broadly used in computational neuroscience. However, these models fail to describe inhibition-based rhythms, typically observed in networks of inhibitory neurons with synaptic kinetics [30–38]. To overcome this limitation, some authors heuristically included explicit delays in traditional FRE, and found qualitative agreement with the oscillatory dynamics observed in simulations of spiking neurons with both synaptic kinetics and fixed time delays [9, 10, 36, 39]. Nonetheless it remains unclear why traditional H-FRE with first order synaptic kinetics do not generate inhibition-based oscillations.

Here we have investigated a novel class of FRE which can be rigorously derived from populations of spiking (QIF) neurons [40]. Networks of globally coupled QIF neurons with fast inhibitory synapses readily generate fast self-sustained oscillations. The corresponding exact FRE, which we call the QIF-FRE, therefore also generates oscillations. The benefit of having a simple macroscopic description for the network dynamics is its amenability to analysis. In particular, the nonlinearities in Eq (3), which arise due to the spike initiation and reset mechanism in the QIF model, conspire to generate damped oscillations which reflect transient spike synchrony in the network. This oscillatory mode can be driven by sufficiently fast recurrent inhibitory synaptic activation, leading to sustained oscillations. This suggests that any mean-field description of network activity which neglects subthreshold integration will not properly capture spike-synchrony-dependent dynamical behaviors, including fast inhibitory oscillations.

The QIF-FRE have also allowed us to generate a phase diagram for oscillatory behavior in a network of QIF neurons with ease via a standard linear stability analysis, see Fig 4. This phase diagram agrees qualitatively with that of an equivalent network of Wang-Buzsáki neurons, suggesting that the QIF-FRE not only provide an exact description of QIF networks, but also a qualitatively accurate description of macroscopic behaviors in networks of Class I neurons in general. In particular, the QIF-FRE capture the fragility of oscillations to quenched variability in the network, a feature that seems to be particularly pronounced for Class 1 neuronal models compared to neural models with so-called Class 2 excitability [59].

Finally we have shown that the QIF-FRE and the heuristic H-FRE are formally equivalent in the limit of slow synapses. In this limit the neuronal dynamics is slaved to the synaptic activation and well-described by Eq (10), as long as external inputs are stationary. In fact, in the absence of quenched heterogeneity (Δ = 0), the approximation for slow synapses Eq (10) is also fully equivalent to the reduction for slow synapses in networks of Class 1 neurons derived by Ermentrout in [60]. This further indicates that the QIF-FRE are likely valid for networks of Class 1 neurons in general. However, we also show that in the more biologically plausible scenario of time-varying external drive some degree of neuronal synchronization is generically observed, as in (Fig 6), and the slow-synapse reduction Eq (10) is not valid.

The results presented here are obtained under two important assumptions that need to be taken into account when comparing our work to the existing literature on fast oscillations in inhibitory networks. (*i*) A derivation of an exact firing rate model for a spiking neuron network is only possible for ensembles of QIF neurons, which are the canonical model for Class 1 excitability [45, 61]. Although many relevant computational studies on fast inhibitory oscillations also consider Class 1 neurons [30, 32, 34, 39, 62–64], experimental evidence indicates that fast spiking interneurons are highly heterogeneous in their minimal firing rates in response to steady currents, and that a significant fraction of them are Class 2 [65–68] —but see also [69]. (*ii*) Our derivation of the QIF-FRE is valid for networks of globally coupled QIF neurons, with Lorentzian-distributed currents. In this system inhibition-based oscillations are only possible when the majority of the neurons are self-sustained oscillators, i.e. for Θ > 0 in Eq (14), and are due to the frequency locking of a fraction of the oscillators in the population [41, 42] —as it can be seen in the raster plot of Fig 1(c). In this state, the frequency of the cluster of synchronized oscillators coincides with the frequency of the mean field. The value of the frequency itself is determined through an interplay between single-cell resonance and network effects. Specifically, the synchronized neurons have intrinsic spiking frequencies near that of the mean-field oscillation and hence are driven resonantly. This collective synchronization differs from the so-called sparse synchronization observed in inhibitory networks of identical Class 1 neurons under the influence of noise [34, 36, 62, 63]. In the sparsely synchronized state neurons fire stochastically at very low rates, while the population firing rate displays the fast oscillations as the ones reported here.

Oscillatory phenomena arising from single-cell resonances, and which reflect spike synchrony at the population level, are ubiquitous in networks of spiking neurons. Mean-field theory for noise-driven networks leading to a Fokker-Planck formalism, allows for a linear analysis of the response of the network to weak stimuli when the network is in an asynchronous state [43, 70]. Resonances can appear in the linear response when firing rates are sufficiently high or noise strength sufficiently low. Recent work has sought to extend H-FRE in this regime by extracting the complex eigenvalue corresponding to the resonance and using it to construct the linear operator of a complex-valued differential equation, the real part of which is the firing rate [29]. Other work has developed an expression for the response of spiking networks to external drive, which often generates resonance-related damped oscillations, through an eigenfunction expansion of the Fokker-Planck equation [71]. Our approach is similar in spirit to such studies in that we also work with a low dimensional description of the network response. In contrast to previous work our equations are an exact description of the macroscopic behavior, although they are only valid for networks of heterogeneous QIF neurons. Nonetheless, the QIF-FRE are simple enough to allow for an intuitive understanding of the origin of fast oscillations in inhibitory networks, and in particular, of why these oscillations are not properly captured by H-FRE.

## Materials and methods

### Populations of inhibitory quadratic integrate and fire neurons

We model fast-spiking interneurons, the dynamics of which are well-described by the Hodgkin-Huxley equations with only standard spiking currents. Many models of inhibitory neurons are Class 1 excitable [72], including for example the Wang-Buszáki (WB) [30], and the Morris-Lecar models [73]. Class 1 models are characterized by the presence of a saddle-node bifurcation on an invariant circle at the transition from quiescence to spiking. One consequence of this bifurcation structure is the fact the spiking frequency can be arbitrarily low near threshold. Additionally, near threshold the spiking dynamics are dominated by the time spent in the vicinity of the saddle-node itself, allowing for a formal reduction in dimensionality from the full neuron model to a reduced normal form equation for a saddle-node bifurcation [2, 45, 61]. This normal form, which is valid for any Class 1 model near threshold, is known as the quadratic integrate-and-fire model (QIF). Using a change of variables, the QIF model can be transformed to a phase model, called Theta-Neuron model [74], which has an strictly positive Phase Resetting Curve (PRC). Neuron models with strictly positive PRC are called Type 1 neurons, indicating that perturbations always produce an advance (and not a delay) of their phase. In general, Class 1 neurons have a Type 1 PRC [45], but see [75, 76].

In a network of QIF neurons, the neuronal membrane potentials are ${\left\{{\tilde{V}}_{i}\right\}}_{i=1,\ldots ,N}$, which obey the following ordinary differential equations [7, 64, 74]:

$$\begin{array}{c} C\frac{d{\tilde{V}}_{i}}{dt}=g_{L}\frac{\left({\tilde{V}}_{i}-V_{t}\right)\left({\tilde{V}}_{i}-V_{r}\right)}{\left(V_{t}-V_{r}\right)}+I_{0,i} \end{array}$$ (11)

where *C* is the cell capacitance, *g*_*L*_ is the leak conductance and *I*_0,*i*_ are external currents. Additionally, *V*_*r*_ and *V*_*t*_ represent the resting potential and threshold of the neuron, respectively. Using the change of variables ${\tilde{V}}_{i}^{\prime }={\tilde{V}}_{i}-\left(V_{t}+V_{r}\right)/2$, and then rescaling the shifted voltages as $V_{i}={\tilde{V}}_{i}^{\prime }/\left(V_{t}-V_{r}\right)$, the QIF model (11) reduces to

$$\begin{array}{c} \tau_{m}{\dot{V}}_{i}=V_{i}^{2}+I_{i} \end{array}$$ (12)

where *τ*_*m*_ = *C*/*g*_*L*_ is the membrane time constant, *I*_*i*_ = *I*_0,*i*_/(*g*_*L*_(*V*_*t*_−*V*_*r*_))−1/4 and the overdot represents derivation with respect to time *t*. Note that in the model (12) the voltage variables *V*_*i*_ and the inputs *I*_*i*_ do not have dimensions. Thereafter we work with QIF model its simplest form Eq (12). We assume that the inputs are

$$\begin{array}{c} I_{i}=\eta_{i}-J\tau_{m}S, \end{array}$$ (13)

where *J* is the inhibitory synaptic strength, and *S* is the synaptic gating variable. Finally, the currents *η*_*i*_ are constants taken from some prescribed distribution that here we consider it is a Lorentzian of half-width Δ, centered at Θ

$$\begin{array}{c} g\left(\eta \right)=\frac{1}{\pi }\frac{\Delta }{{\left(\eta -\Theta \right)}^{2}+\Delta^{2}}. \end{array}$$ (14)

In numerical simulations the currents were selected deterministically to represent the Lorentzian distribution as: *η*_*i*_ = Θ + Δtan(*π*/2(2*i* − *N* − 1)/(*N* + 1)), for *i* = 1, …, *N*. In the absence of synaptic input, the QIF model Eqs (12) and (13) exhibits two possible dynamical regimes, depending on the sign of *η*_*i*_. If *η*_*i*_ < 0, the neuron is excitable, and an initial condition $V_{i}\left(0\right)<\sqrt{-\eta_{i}}$, asymptotically approaches the resting potential $-\sqrt{-\eta_{i}}$. For initial conditions above the excitability threshold, $V_{i}\left(0\right)>\sqrt{-\eta_{i}}$, the membrane potential grows without bound. In this case, once the neuron reaches a certain threshold value *V*_*θ*_ ≫ 1, it is reset to a new value −*V*_*θ*_ after a refractory period 2*τ*_*m*_/*V*_*θ*_ (in numerical simulations, we choose *V*_*θ*_ = 100). On the other hand, if *η*_*j*_ > 0, the neuron behaves as an oscillator and, if *V*_*θ*_ → ∞, it fires regularly with a period $T=\pi \tau_{m}/\sqrt{\eta_{i}}$. The instantaneous population mean firing rate is

$$\begin{array}{c} R=\lim_{\tau_{s}\to 0}\frac{1}{N}\frac{1}{\tau_{s}}\sum_{j=1}^{N}\sum_{k}\int_{t-\tau_{s}}^{t}dt^{\prime }\delta \left(t^{\prime }-t_{j}^{k}\right), \end{array}$$ (15)

where $t_{j}^{k}$ is the time of the *k*th spike of *j*th neuron, and *δ*(*t*) is the Dirac delta function. Finally, the dynamics of the synaptic variable obeys the first order ordinary differential equation

$$\begin{array}{c} \tau_{d}\dot{S}=-S+R. \end{array}$$ (16)

For the numerical implementation of Eqs (15) and (16), we set *τ*_*s*_ = 10^−2^ *τ*_*m*_. To obtain a smoother time series, the firing rate plotted in Fig 3 was computed according to Eq (15) with *τ*_*s*_ = 3 ⋅ 10^−2^ *τ*_*m*_.

### Firing rate equations for populations of quadratic integrate and fire neurons

Recently Luke et al. derived the exact macroscopic equations for a pulse-coupled ensemble of Theta-Neurons [77], and this has motivated a considerable number of recent papers [78–86, 88]. This work applies the so-called Ott-Antonsen theory [89–91] to obtain a low-dimensional description of the network in terms of the complex Kuramoto order parameter. Nevertheless, it is is not obvious how these macroscopic descriptions relate to traditional H-FRE.

As we already mentioned, the Theta-neuron model exactly transforms to the Quadratic Integrate and Fire (QIF) model by a nonlinear change of variables [45, 61, 74]. This transformation establishes a map between the phase variable *θ*_*i*_ ∈ (−*π*, *π*] of a Theta neuron *i*, and the membrane potential variable *V*_*i*_ ∈ (−∞, +∞) of the QIF model Eq (12). Recently it was shown that, under some circumstances, a change of variables also exists at the population level [40]. In this case, the complex Kuramoto order parameter transforms into a novel order parameter, composed of two macroscopic variables: The population-mean membrane potential *V*, and the population-mean firing rate *R*. As a consequence of that, the Ott-Antonsen theory becomes a unique method for deriving exact firing rate equations for ensembles of heterogeneous spiking neurons —see also [92–94] for recent alternative approaches.

Thus far, the FRE for QIF neurons (QIF-FRE) have been successfully applied to investigate the collective dynamics of populations of QIF neurons with instantaneous [40, 86, 87], time delayed [95] and excitatory synapses with fast synaptic kinetics [96]. However, to date the QIF-FRE have not been used to explore the dynamics of populations of inhibitory neurons with synaptic kinetics —but see [83] for a numerical investigation using the low-dimensional Kuramoto order parameter description. The method for deriving the QIF-FRE corresponding to a population of QIF neurons Eq (12) is exact in the thermodynamic limit *N* → ∞, and, under the assumption that neurons are all-to-all coupled. Additionally, if the parameters *η*_*i*_ in Eq (13) (which in the thermodynamic limit become a continuous variable) are assumed to be distributed according to the Lorentzian distribution Eq (14), the resulting QIF-FRE become two dimensional. For instantaneous synapses, the macroscopic dynamics of the population of QIF neurons (12) is exactly described by the system of QIF-FRE [40]

$$\begin{array}{ccc} \tau_{m}\dot{R} & = & \frac{\Delta }{\pi \tau_{m}}+2RV, \end{array}$$ (17a)

$$\begin{array}{ccc} \tau_{m}\dot{V} & = & V^{2}-{\left(\pi \tau_{m}R\right)}^{2}-J\tau_{m}R+\Theta , \end{array}$$ (17b)

where *R* is the mean firing rate and *V* the mean membrane potential in the network. With exponentially decaying synaptic kinetics the QIF-FRE Eq (17) become Eq (3). In our study, we consider Θ > 0, so that the majority of the neurons are oscillatory —see Eq (14).

#### Fixed points

The fixed points of the QIF-FRE (3) are obtained imposing $\dot{R}=\dot{V}=\dot{S}=0$. Substituting this into Eq (3), we obtain the fixed point equation V* = −Δ/(2*πτ*_*m*_ R*), the firing rate given by Eq (4) and S_*_ = R_*_. Note that for homogeneous populations, Δ = 0, the f-I curve Eq (5) reduces to

$$\begin{array}{c} \Phi \left(I\right)=\frac{1}{\pi }\sqrt{{\left|I\right|}_{+}}, \end{array}$$

which displays a clear threshold at *I* = 0 (Here, |*I*|_+_ = *I* if *I* ≥ 0, and vanishes for *I* < 0.) This function coincides with the *squashing function* found by Ermentrout for homogeneous networks of Class 1 neurons [60]. As expected, for heterogeneous networks, the well-defined threshold of Φ(*I*) for Δ = 0 is lost and the transfer function becomes increasingly smoother.

#### Nondimensionalized QIF-FRE

The QIF-FRE (3) have five parameters. It is possible to non-dimensionalize the equations so that the system can be written solely in terms of 3 parameters. Generally, we adopt the following notation: we use capital letters to refer to the original variables and parameters of the QIF-FRE, and lower case letters for non-dimensional quantities. A possible non-dimensionalization, valid for Θ > 0, is

$$\dot{r}=\delta /\pi +2rv,$$ (18a)

$$\dot{v}=v^{2}-\pi^{2}r^{2}-js+1,$$ (18b)

$$\tau \dot{s}=-s+r,$$ (18c)

where the overdot here means differentiation with respect to the non-dimensional time

$$\begin{array}{c} \tilde{t}=\frac{\sqrt{\Theta }}{\tau_{m}}t. \end{array}$$

The other variables are defined as

$$\begin{array}{c} r=\frac{\tau_{m}}{\sqrt{\Theta }}R,\;v=\frac{V}{\sqrt{\Theta }},\;s=\frac{\tau_{m}}{\sqrt{\Theta }}S. \end{array}$$

On the other hand, the new coupling parameter is defined as

$$\begin{array}{c} j=\frac{J}{\sqrt{\Theta }}. \end{array}$$ (19)

and the parameter

$$\begin{array}{c} \delta =\frac{\Delta }{\Theta }, \end{array}$$ (20)

describes the effect of the Lorentzian heterogeneity (14) into the collective dynamics of the FRE (17). Though the Lorentzian distribution does not have finite moments, for the sake of comparison of our results with those of studies investigating the dynamics of heterogeneous networks of inhibitory neurons, e.g. [30, 32], the quantity *δ* can be compared to the coefficient of variation, which measures the ratio of the standard deviation to the mean of a probability density function. Finally, the non-dimensional time

$$\begin{array}{c} \tau =\frac{\sqrt{\Theta }}{\tau_{m}}\tau_{d}, \end{array}$$ (21)

measures the ratio of the synaptic time constant to the most-likely period of the neurons (times *π*),

$$\begin{array}{c} \bar{T}=\pi \frac{\tau_{m}}{\sqrt{\Theta }}. \end{array}$$

In numerical simulations we will use the original QIF-FRE (3), with Θ = 4, and *τ*_*m*_ = 10ms. Thus $\bar{T}=10\pi /3\approx 15.71\;\text{ms}$, so that the most likely value of the neurons’ intrinsic frequency is $\bar{f}\approx 63.66\;\text{Hz}$. However, our results are expressed in a more compact form in terms of the quantities *j*, *δ*, *τ*, and we will use them in some of our calculations and figures.

### Parametric formula for the Hopf boundaries

To investigate the existence of oscillatory instabilities we use Eq (6) written in terms of the non-dimensional variables and parameters defined previously, which is

$$\begin{array}{c} -2jr_{*}=\left(1+\tilde{\lambda }\tau \right)\left[{\left(2\pi r_{*}\right)}^{2}+{\left(\tilde{\lambda }+\frac{\delta }{\pi r_{*}}\right)}^{2}\right]. \end{array}$$ (22)

Imposing the condition of marginal stability $\tilde{\lambda }=i\tilde{\omega }$ in Eq (22) gives the system of equations

$$\begin{array}{ccc} 0 & = & 2jr_{*}+4\pi^{2}r_{*}^{2}+4v_{*}^{2}-\left(1-4v_{*}\tau \right){\tilde{\omega }}^{2} \end{array}$$ (23a)

$$\begin{array}{ccc} 0 & = & \tilde{\omega }\left(4v_{*}-4\pi^{2}r_{*}^{2}\tau -4v_{*}^{2}\tau +\tau {\tilde{\omega }}^{2}\right) \end{array}$$ (23b)

where the fixed points are obtained from Eq (4) solving

$$\begin{array}{c} 0=v_{*}^{2}-\pi^{2}r_{*}^{2}-jr_{*}+1, \end{array}$$ (24)

with

$$\begin{array}{c} v_{*}=-\frac{\delta }{2\pi r_{*}} \end{array}$$

Eq (23b) gives the critical frequency

$$\begin{array}{c} \tilde{\omega }=\frac{2}{\tau }\sqrt{{\left(\pi \tau r_{*}\right)}^{2}+\tau v_{*}\left(\tau v_{*}-1\right)}. \end{array}$$

The Hopf boundaries can be plotted in parametric form solving Eq (24) for *j*, and substituting *j* and $\tilde{\omega }$ into Eq (23a). Then solving Eq (23a) for *τ* gives the Hopf bifurcation boundaries

$$\begin{array}{c} \tau^{\pm }\left(r_{*}\right)=\frac{\pi^{2}r_{*}^{2}-1+7v_{*}^{2}\pm \sqrt{{\left(\pi^{2}r_{*}^{2}-1\right)}^{2}-\left(14+50\pi^{2}r_{*}^{2}\right)v_{*}^{2}-15v_{*}^{4}}}{16v_{*}\left(\pi^{2}r_{*}^{2}+v_{*}^{2}\right)}. \end{array}$$ (25)

Using the parametric formula

$$\begin{array}{c} {\left(j\left(r_{*}\right),\tau^{\pm }\left(r_{*}\right)\right)}^{\pm }=\left(v_{*}^{2}/r_{*}+1/r_{*}-\pi^{2}r_{*},\tau^{\pm }\left(r_{*}\right)\right). \end{array}$$

we can be plot the Hopf boundaries for particular values of the parameter *δ*, as r_*_ is changed. Fig 4 shows these curves in red, for *δ* = 0.05 and *δ* = 0.075. They define a closed region in parameter space (shaded region) where oscillations are observed.

#### Calculation of the critical value *δ*_*c*_, Eq (8)

The functions *τ*^±^ meet at two points, when the argument of the square root in Eq (25) is zero. This gives four different roots for *δ*, and only one of them is positive and real

$$\begin{array}{c} \delta^{*}\left(r_{*}\right)=\frac{2\pi r_{*}}{\sqrt{15}}\sqrt{8\sqrt{1+5\pi^{2}r_{*}^{2}+10\pi^{4}r_{*}^{4}}-7-25\pi^{2}r_{*}^{2}}. \end{array}$$

This function has two positive zeros, one at r_*min_ = 0, and one at r_*max_ = 1/*π*, corresponding, respectively, to the minimal (*j* → ∞) and maximal (*j* = 0) values of the firing rate for identical neurons (*δ* = 0). Between these two points the function attains a maximum, where r_*min_ = r_*max_ = r_*c_, with

$$\begin{array}{c} r_{*c}=\frac{1}{\sqrt{2\sqrt{5}}\pi }=0.1505\ldots \end{array}$$

The function δ*(r_*_) evaluated at its local maximum r_*_ = r_*c_ gives Eq (8).

### Populations of Wang-Buzsáki neurons

We perform numerical simulations using the the Wang-Buzsáki (WB) neuron [30], and compare them with our results using networks of QIF neurons. The onset of oscillatory behavior in the WB model is via a saddle node on the invariant circle (SNIC) bifurcation. Therefore, the populations of WB neurons near this bifurcation are expected to be well described by the theta-neuron/QIF model, the canonical model for Class 1 neural excitability [45, 74].

We numerically simulated a network of *N* all-to-all coupled WB neurons, where the dynamics of each neuron is described by the time evolution of its membrane potential [30]

$$\begin{array}{c} C_{m}\dot{V_{i}}=-I_{\text{Na},i}-I_{\text{K},i}-I_{\text{L},i}-I_{\text{syn}}+I_{\text{app},i}+I_{0}. \end{array}$$

The cell capacitance is *C*_*m*_ = 1 *μ*F/cm^2^. The inputs *I*_app_ (in *μ*A/cm^2^) are distributed according to a Lorentzian distribution with half width *σ* and center $\bar{I}$. In numerical simulations these currents were selected deterministically to represent the Lorentzian distribution as $I_{\text{app},i}=\bar{I}+\sigma \tan\left(\pi /2\left(2i-N-1\right)/\left(N+1\right)\right)$, for *i* = 1, …, *N*. The constant input *I*_0_ = 0.1601 *μ*A/cm^2^ sets the neuron at the SNIC bifurcation when *I*_*app*_ = 0. The leak current is

$$\begin{array}{c} I_{\text{L},i}=g_{\text{L}}(V_{i}-E_{\text{L}}), \end{array}$$

with *g*_L_ = 0.1 mS/cm^2^, so that the passive time constant *τ*_*m*_ = *C*_*m*_/*g*_L_ = 10 ms. The sodium current is

$$\begin{array}{c} I_{\text{Na},i}=g_{\text{Na}}m_{\infty }^{3}h(V_{i}-E_{\text{Na}}), \end{array}$$

where *g*_Na_ = 35 mS/cm^2^, *E*_Na_ = 55 mV, *m*_∞_ = *α*_*m*_/(*α*_*m*_ + *β*_*m*_) with *α*_*m*_(*V*_*i*_) = −0.1(*V*_*i*_ + 35)/(exp(−0.1(*V*_*i*_ + 35) − 1)), *β*_*m*_(*V*_*i*_) = 4exp(−(*V*_*i*_ + 60)/18). The inactivation variable *h* obeys the differential equation

$$\begin{array}{c} \dot{h}=\phi (\alpha_{h}(1-h)-\beta_{h}h), \end{array}$$

with *ϕ* = 5, *α*_*h*_(*V*_*i*_) = 0.07exp(−(*V*_*i*_ + 58)/20) and *β*_*h*_(*V*_*i*_) = 1/(exp(−0.1(*V*_*i*_ + 28)) + 1). The potassium current follows

$$\begin{array}{c} I_{\text{K},i}=g_{\text{K}}n^{4}(V_{i}-E_{\text{K}}), \end{array}$$

with *g*_K_ = 9 mS/cm^2^, *E*_K_ = −90 mV. The activation variable *n* obeys

$$\begin{array}{c} \dot{n}=\phi (\alpha_{n}(1-n)-\beta_{n}n), \end{array}$$

where *α*_*n*_(*V*_*i*_) = −0.01(*V*_*i*_ + 34)/(exp(−0.1(*V*_*i*_ + 34)) − 1) and *β*_*n*_(*V*_*i*_) = 0.125exp(−(*V*_*i*_ + 44)/80).

The synaptic current is *I*_syn_ = *kC*_*m*_ *S*, where the synaptic activation variable *S* obeys the first order kinetics Eq (16) and *k* is the coupling strength (expressed in mV). The factor *C*_*m*_ ensures that the effect of an incoming spike to the neuron is independent from its passive time constant. The neuron is defined to emit a spike when its membrane potential crosses 0 mV. The population firing rate is then computed according to Eq (15), with *τ*_*s*_ = 10^−2^ ms. In numerical simulations we considered *N* = 1000 all-to-all coupled WB neurons, using the Euler method with time step *dt* = 0.001 ms. In Fig 1, the membrane potentials were initially randomly distributed according to a Lorentzian function with half width 5 mV and center −62 mV. Close to the bifurcation point, this is equivalent to uniformly distribute the phases of the corresponding Theta-Neurons in [−*π*, *π*] [2, 7, 61, 74]. The parameters were chosen as $\bar{I}=0.5\;\mu \text{A}/{\text{cm}}^{2}$, *σ* = 0.01 *μ*A/cm^2^ and *k* = 6 mV. The population firing rate was smoothed setting *τ*_*s*_ = 2 ms in Eq (15).

In Fig 5, we systematically varied the coupling strength and the synaptic time decay constant to determine the range of parameters displaying oscillatory behavior. For each fixed value of *τ*_*d*_ we varied the coupling strength *k*; we performed two series of simulations, for increasing and decreasing coupling strength. In Fig 5 we only show results for increasing *k*.

All quantities were measured after a transient of 1000 ms. To obtain the amplitude of the oscillations of the mean membrane potential, we computed the maximal amplitude ${\bar{V}}_{\text{max}}-{\bar{V}}_{\text{min}}$ over time windows of 200 ms for 1000 ms, and then averaged over the five windows.
